# Acute Complications of United States Service Members with Combat-Related Lower Extremity Limb Salvage

**DOI:** 10.3390/jcm14113923

**Published:** 2025-06-03

**Authors:** Susan L. Eskridge, Benjamin Huang, Aidan McQuade, Stephen M. Goldman, Christopher L. Dearth

**Affiliations:** 1 Leidos, San Diego, CA 20190, USA; 2 Department of Surgery, Uniformed Services University of the Health Sciences, Bethesda, MD 20814, USA; 3 Extremity Trauma and Amputation Center of Excellence, Defense Health Agency, Falls Church, VA 22042, USA

**Keywords:** military medicine, trauma, orthopedics, limb loss, reconstructive surgery

## Abstract

**Background**: This study examined the incidence of acute complications within the first year following combat-related lower extremity injuries in United States (U.S.) Service members (SMs). The research compared outcomes between primary amputation (PA), limb salvage (LS), and non-threatening limb trauma (NTLT) groups, and conducted a subgroup analysis within the LS group, differentiating between SM who underwent a secondary amputation (LS-SA) and those who did not (LS-NA). **Methods**: A retrospective analysis of combat-related lower extremity injuries sustained between January 2001 and October 2015 was performed using data from the Military Health System Medical Data Repository. Chi-square tests and adjusted logistic regression analysis were used to compare complication frequencies by injury severity. **Results***:* The analysis of the 4275 SM revealed that 21% had undergone PA, 47% LS (with 13% experiencing LS-SA and 87% LS-NA), and NTLT was observed in 32% of cases. The PA group exhibited higher rates of most acute complications compared to other groups, with three exceptions—i.e., non-union fractures, compartment syndrome, and orthopedic device complications were more prevalent in the LS group than the PA group. The LS-SA group had higher complication rates than the LS-NA group for most complications. Notably, the PA group was associated with the highest rates of post-hemorrhagic anemia and heterotopic ossification, while the LS-SA group exhibited the highest rates of osteomyelitis, non-union fractures, non-healing wounds, and compartment syndrome. **Conclusions**: Individuals with amputation (PA or LS-SA) were more likely to experience acute complications compared to their counterparts (PA vs. LS and NTLT; LS-SA vs. LS-NA), with the exception of non-union fractures, which were more frequent in the LS group than the PA group. These findings highlight the need for close monitoring and targeted interventions to address post-surgical complications in Service members with limb salvage.

## 1. Introduction

During the conflicts in Iraq and Afghanistan, musculoskeletal trauma was highly prevalent, with over half of all injuries involving the extremities [1]. This large volume of complex orthopedic injuries is believed to be due to the increased use of improvised explosive devices and improved body armor for Service members (SMs) and vehicles [2]. Consequently, more critically injured combat casualties survived to receive definitive surgical care due to the increased use of tourniquets, blood transfusions, and advanced prehospital care systems [3].

The most severe extremity trauma cases often result in immediate amputation(s) or extensive reconstructive procedures. The severity and complexity of these injuries have raised questions not only regarding whether or not to amputate the injured limb but also about the acute and long-term consequences of these injuries. Advancements in multidisciplinary surgical fields have increased both the desire and ability to salvage critically injured limbs to restore function and have revealed the need for further research on subsequent complications to fully care for traumatic extremity injuries [4,5].

A 2017 retrospective study analyzed four-year health outcomes in lower extremity combat-injured patients with early amputations, late amputations, or limb salvage. The study offered valuable insights into the physical and psychological pathologies that occurred as a direct result of combat-related extremity trauma [6]. However, it is important to note that limb salvage (LS) procedures were defined as one of more of the following: complex fractures (Gustilo–Anderson classification for open fractures: grades IIIC, IIIB, and selected IIIA), vascular injuries, major soft tissue injuries, and/or severe foot injuries; this is because there was no widely accepted definition to identify the limb salvage population at the time. While this approach was common amongst many studies at the time it is inherently prone to oversimplification as the combination of seemingly less severe injuries could in aggregate threaten the limb and be included in a limb salvage definition. Moreover, a priori definitions are often based on the collective experience and opinions of experts at a specific point in time. While valuable, this can introduce biases based on their training, institutional practices, and the specific patient populations they have encountered. Subsequently, a particular set of ICD−9-CM codes has been established through a data-driven process to identify the population of patients who underwent combat-related LS in military health system databases [7]. Briefly, this method involved analyzing data from Service members (SMs) with lower extremity trauma, comparing those who underwent secondary amputations (used as a surrogate for LS cases) with those who did not. A combination of “OR” and “AND” gating strategies was used to join significant diagnosis and procedure codes, defining LS as cases with both a diagnosis and a procedure significantly associated with the surrogate population. The code set was validated against the consensus of military trauma surgeons, revealing 70% agreement and a moderate Kappa statistic (κ = 0.55), with the data-driven method demonstrating 55.6% sensitivity and 87% specificity [7].

This study analyzes a more complete, data-driven study population [8], quantifying the most common acute complications within the first year after injury and comparing those across several extremity trauma cohorts. Our conclusions emphasize the importance of accurately defining injury severity groups in combat trauma, particularly concerning LS. Extremity injuries sustained in combat significantly impact SM careers and quality of life. As research and surgical techniques progress, understanding the most common acute complications across different limb trauma severity levels will guide future care to mitigate potential complications.

## 2. Methods

This study, approved by the Naval Health Research Center Institutional Review Board (Protocol# NHRC.2003.0025, approved: 1 October 2003), investigated acute complications one year following combat-related lower extremity injuries sustained between 1 January 2001 and 31 October 2015. The research compared these complications across three injury severity cohorts: primary amputation (PA), LS, and non-threatening limb trauma (NTLT). Furthermore, a sub-analysis of the LS cohort compared complications between those who subsequently underwent secondary amputation (LS-SA) and those with no recorded history of amputation (LS-NA).

The study sample and data source for injury information were described previously [7,8]. The Military Health System Medical Data Repository (MDR) was linked to the study sample to obtain medical diagnosis records within the first year after injury. Cases without an MDR record were excluded from the study. The acute complications of interest were determined by previous research [6,9] and the authors’ expertise to examine a broad set of complications. These complications were identified within the first year post-injury using ICD−9-CM codes from both inpatient and outpatient MDR records. The analysis focused on acute complications with at least 5% prevalence in the study population.

The prevalence of acute complications is presented by group category as frequency and percent. Chi-square tests were used to compare the frequency of each acute complication across injury groups examining PA, LS, and NTLT separately from LS-SA and LS-NA. The incidence of the acute complications was determined by new cases of each complication in the population for each quarter (every three months) during the first year. Chi-square tests were used to compare the frequency of each acute complication across the injury groups (PA, LS, and NTLT) for each quarter separately. Multivariate logistic regression was used to examine the association between each acute complication outcome with injury group as the independent variable. Odds ratios (ORs) and 95% confidence intervals (95% CIs) as well as *p*-values were reported for PA and LS with NTLT as the reference group, as well as LS-SA with LS-NA as the reference group. The covariates (age, mechanism of injury, polytrauma designation) were determined a priori and have been reported previously in combat-related extremity trauma [2].

## 3. Results

Among the 4,275 SM, 885 (21%) sustained PA, 2,018 (47%) underwent initial LS procedures, and 1,372 (32%) were non-threatening limb trauma. Within the LS cohort, 269 (13%) underwent a subsequent secondary amputation procedure (LS-SA), whereas 1,749 (87%) did not (LS-NA). The PA group experienced higher rates of acute complications compared to the LS and NTLT groups, except for three of the complications examined (Table 1).

The complications that were higher in the LS group compared to the PA group were non-union fracture (PA, 9.5%; LS, 20.4%; NTLT, 6.6%); compartment syndrome (PA, 5.8%; LS, 13.0%; NTLT, 4.8%), and mechanical complications of an orthopedic device (PA, 6.1%; LS, 8.2%; NTLT, 2.3%). When comparing the LS groups (Table 2), the LS-SA group had higher rates than the LS-NA group in all complications except for deep vein thrombosis in the lower extremity (5.9% vs. 5.7%). Notably, the PA group had the highest rates of acute post-hemorrhagic anemia (65.3%) and heterotopic ossification (39.4%), with the LS-SA group having the highest rates of osteomyelitis (41.6%), non-union fracture (32.0%), non-healing wounds (23.0%), and compartment syndrome (20.8%).

**Figure 1:**
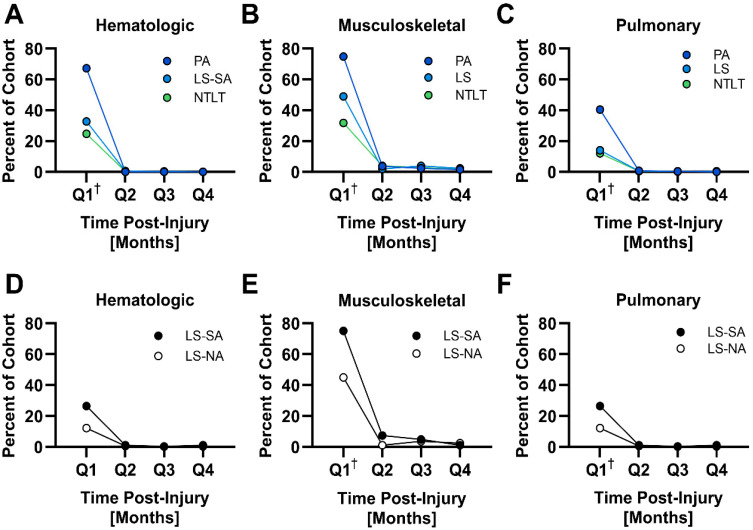
Quarterly incidence of acute complications by biological system during the first year after injury. This figure presents line graphs illustrating the incidence of acute complications over four quarters following injury, categorized by the affected biological system. A dagger symbol (†) denotes a higher incidence in that quarter compared to all other quarters for a given biological system (*p* < 0.05).

To analyze the incidence of complications, they were grouped by the biological system affected. The occurrence of complications within each group was then assessed quarterly during the first year to determine the timing of diagnoses. The majority of the complication groupings exhibited the highest incidence rates in the first quarter, with a subsequent drop-off in rates. For example, hematologic complications had incidence rates ranging from 24.7% to 67.23% in the first quarter to less than 1% across all groups in the second quarter (Figure 1). Complications impacting the musculoskeletal and pulmonary systems followed similar trends.

As it relates to these specific complications, a few notable exceptions to this pattern were observed. Both LS-SA and LS-NA groups exhibited a higher incidence of non-union fracture in the second quarter than in the first quarter and relatively sustained rates throughout the year (LS-SA: first quarter, 8%; second quarter, 11%; third quarter, 8%; fourth quarter, 5%. LS-NA: first quarter, 5%; second quarter, 7%; third quarter, 4%; fourth quarter, 3%). Relatively sustained rates during the first year were also observed in SM with PA for heterotopic ossification (first quarter, 15%; second quarter, 10%; third quarter, 9%; fourth quarter, 4%) as well as for complications with orthopedic devices (first quarter, 6%; second quarter, 3%; third quarter, 3%; fourth quarter, 3%) in SM with LS-SA.

**Figure 2:**
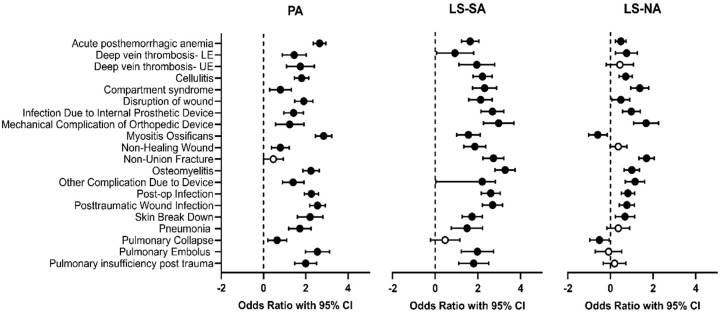
Forest plots displaying odds ratios (ORs) and their corresponding 95% confidence intervals for the development of highly prevalent complications. PA, LS-SA, and LS-NA were compared to NTLT. Data points colored in black indicate *p* < 0.05.

Multivariate logistic regression was carried out to examine the three injury groups (PA, LS, and NTLT) with the limb salvage subgroups (LS-SA and LS-NA) broken out separately, including adjustments for age, mechanism of injury, and polytrauma (Figure 2). There were higher odds for most of the diagnoses examined in PA, LS-SA, and LS-NA compared to NTLT. The LS-SA group exhibits higher odds of experiencing every diagnosis examined, with the exception of pulmonary collapse. The LS-NA group exhibited higher odds of all complications except myositis ossificans, deep vein thrombosis of the upper extremity, and the four pulmonary complications. In fact, myositis ossificans and pulmonary collapse exhibited reduced odds relative to NTLT.

The complications where the odds were higher in the PA group than in the LS group were acute post-hemorrhagic anemia, disruption of wound, heterotopic ossification, osteomyelitis, cellulitis, post-traumatic wound infection, postoperative infection, and all four of the pulmonary complications. The only complication where the odds were higher in the LS group than in the PA group was non-union fracture. The odds of acute complications in the LS-SA group were all higher than in the LS-NA group, except for deep vein thrombosis in the lower extremity, where there was no association with injury group. The highest odds of acute complications in SM with LS-SA compared to LS-NA were with osteomyelitis, myositis ossificans, and pulmonary embolism.

## 4. Discussion

This study investigated the frequency and odds of acute complication diagnoses among three lower limb injury groups (PA, LS, and NTLT). The analysis also compared complication rates between individuals with LS who did or did not undergo secondary amputation (LS-SA vs. LS-NA). Although differences in how studies choose to define limb salvage limit direct comparison with prior studies, our findings reflect some interesting alignments and contrasts with the extant literature. In our study, the rates of acute complication diagnoses were highest in the PA and LS-SA groups. Specifically, individuals with PA consistently exhibited higher odds of most acute complication diagnoses compared to those with LS or NTLT. This finding contradicts previous studies, which have generally shown that limb salvage is associated with a greater risk of complications and subsequent hospital readmissions [9,10,11,12,13]. Although individuals with LS in our study showed increased odds for most complications compared to the NTLT group, these differences were generally marginal relative to the NTLT group, particularly among those in our LS-NA cohort. Interestingly, the LS-SA cohort more closely resembled our PA cohort than the LS-NA cohort. The LS-SA cohort experienced higher rates of all acute complications compared to LS-NA, with the exception of deep vein thrombosis of the lower limb.

Our study’s findings are consistent with the extant literature in two key areas: higher complication rates were observed in amputation groups compared to the limb salvage (LS) group, and non-union fractures were prevalent within our LS cohort.

Specifically, our results align with those of Melcer et al. (2013) [14]. Similar to their report, our amputation patients experienced significantly higher rates of anemia, infections, and heterotopic ossification (HO). Furthermore, our patients who underwent late amputation (classified as LS-SA: limb salvage converted to secondary amputation) generally had higher rates of infectious complications and non-healing wounds compared to our early amputation (EA) or LS patients, which mirrors findings for comparable groups in the Melcer study.

It is crucial to note that these similarities emerge despite differences in how the patient groups were defined. Melcer et al.’s ‘primary amputation’ group included patients amputated within 15 days, whereas our EA group had a 90-day window. Their ‘late amputation’ group (>90 days) corresponds to our LS-SA classification. Additionally, their ‘limb salvage’ group is equivalent to our LS-NA group (limb salvage not amputated).

Our finding that the LS-SA group exhibited the highest frequencies of non-union fractures, osteomyelitis, and non-healing wounds is also consistent with a follow-up paper by Melcer et al. (2017) [6], which reported similarly high rates for these specific complications in their comparable late amputation cohort.

In a similar vein, Ozmen et al. [15] reported non-union rates of ~16% in Type IIIC lower extremity fracture reconstruction cases, while Huh et al. [12] reported that patients definitively managed with late amputation were more likely to have their limb salvage course complicated by infection, citing a 54.5% prevalence of osteomyelitis, findings that are agreeable with the observations in our LS and LS-SA populations, respectively. Harris et al. [9] reported high rates of non-union fractures and osteomyelitis in their limb reconstruction group. While their amputation group had higher infection rates than the limb reconstruction group, the absolute prevalence of infection in their limb reconstruction group was comparable to our LS cohort. Interestingly, Harris et al. indicated that complications peaked at 6 months post-injury, suggesting a potential delay in complication onset compared to our LS groups. This difference could be attributed to various factors, including disparities in healthcare access. Furthermore, Harris et al. reported a notably higher rate of non-union in their limb reconstruction cohort. Their rate of 31% was approximately 1.5 times higher than what we observed in our study cohort, highlighting a potential link between presentation of complications and fracture healing outcomes [9]. Despite potential variations in injury group definitions, the consistency across these studies emphasizes the need for targeted interventions to improve outcomes in this population, particularly regarding non-healing fractures, wounds, and infections.

## 5. Limitations

This study’s limitations include the retrospective design, notably one that relies on the accuracy of electronic medical records. Generalizability is also limited, as the study focuses on a specific population of Service members with combat-related lower extremity injuries. Therefore, the findings may not be directly applicable to civilians or individuals with injuries to other body regions. The most significant difference between civilian and combat-related trauma lies in the mechanisms of injury. Combat-related trauma often involves high-energy mechanisms (e.g., blast, gunshot wounds) that result in a high degree of tissue destruction and contamination, while civilian trauma has a higher prevalence of motor vehicle accidents, falls, and industrial accidents [16]. Furthermore, this study focuses on a specific population of Service members. This demographic is generally younger, more physically fit, and may have different baseline health statuses compared to the diverse age range and varying health conditions seen in civilian trauma populations [17]. As such, their physiological reserve and response to injury and treatment could differ [18]. It is important to note that acute complications can occur at any point during the first year post-injury, a period when patients may still be receiving ongoing care. Consequently, complications could arise before or after secondary amputation in the LS-SA group, or be related to the treatment or sequelae of other injuries. However, the multivariate analysis controlled for polytrauma, mitigating the potential influence of additional injuries on the results. Furthermore, the validity of our method for defining the LS and NTLT groups is supported by the expected correlation between injury severity and acute complications, as well as the consistency of our findings with other research using similar classifications.

## 6. Conclusions

In conclusion, individuals with amputation (PA or LS-SA) were more likely to experience acute complications compared to their counterparts (PA vs. LS and NTLT; LS-SA vs. LS-NA), with the exception of non-union fractures, which were more frequent in the LS group than the PA group. These findings underscore the necessity of close monitoring and targeted interventions to manage post-surgical complications in Service members with extremity trauma. In particular, improved monitoring and early detection of infections could enable earlier intervention and mitigation of adverse outcomes, especially among the limb salvage cohort, given the significant association between complications and limb retention outcomes. Consequently, effectively preventing or resolving these complications may reduce the need for subsequent revision surgeries and/or elective amputations, potentially leading to a smoother recovery and improved functional outcomes, as well as enhancing the ability to return to military service. As such, further research into sensors and biomarkers that could signal the onset of complications, especially during the initial month post-injury, is crucial.

## Figures and Tables

**Table 1:** Acute outcome diagnoses by lower extremity trauma cohort designation.

Acute Outcomes	PA n = 885		LS n = 2018		NTLT n = 1372		Holm-Šídák Adjusted *p*-Values
	*f*	%	*f*	%	*f*	%	
**Hematologic**							
Deep vein thrombosis—UE	69	7.8	76	3.8	35	2.5	<0.001
Deep vein thrombosis—LE	85	9.6	116	5.7	51	3.7	<0.001
Acute post-hemorrhagic anemia	578	65.3	617	30.6	317	23.1	<0.001
**Musculoskeletal/Integumentary**							
Disruption of wound	175	19.8	196	9.7	82	6.0	<0.001
Non-healing wound	123	13.9	232	11.5	105	7.6	<0.001
Skin breakdown	155	17.5	177	8.8	66	4.8	<0.001
Myositis ossificans	349	39.4	139	6.9	100	7.3	<0.001
Osteomyelitis	243	27.5	341	16.9	96	7.0	<0.001
Non-union fracture	84	9.5	411	20.4	91	6.6	<0.001
Compartment syndrome	75	8.5	262	13.0	66	4.8	<0.001
Mechanical complication of orthopedic device	54	6.1	165	8.2	32	2.3	<0.001
Other complication due to device	100	11.3	200	9.9	58	4.2	<0.001
Infection due to internal prosthetic device	130	14.7	243	12.0	70	5.1	<0.001
Cellulitis	284	32.1	388	19.2	147	10.7	<0.001
Post-traumatic wound infection	301	34.0	317	15.7	111	8.1	<0.001
Post-op infection	325	36.7	407	20.2	146	10.6	<0.001
**Pulmonary**							
Pulmonary embolus	142	16.0	75	3.7	41	3.0	<0.001
Pneumonia	128	14.5	111	5.5	49	3.6	<0.001
Pulmonary insufficiency post-trauma	162	18.3	114	5.6	56	4.1	<0.001
Pulmonary collapse	122	13.8	116	5.7	94	6.8	<0.001

Notes: Frequencies represent the number of Service members with at least one of the indicated diagnoses. Abbreviations: PA—primary amputation; LS—limb salvage; NTLT—non-threatening limb trauma.

**Table 2:** Acute outcome diagnoses by limb salvage subgroup.

Acute Outcome Categories and Diagnoses	LS-SA n = 269		LS-NA n = 1749		Holm–Šídák Adjusted *p*-Values
	*f*	%	*f*	%	
**Hematologic**					
Deep vein thrombosis—UE	21	7.8	55	3.1	0.001
Deep vein thrombosis—LE	16	5.9	100	5.7	0.109
Acute post-hemorrhagic anemia	122	45.3	495	28.3	<0.001
**Musculoskeletal/Integumentary**					
Disruption of wound	56	20.8	140	8.0	<0.001
Non-healing wound	62	23.0	170	9.7	<0.001
Skin breakdown	49	18.2	128	7.3	<0.001
Myositis ossificans	51	19.0	88	5.0	<0.001
Osteomyelitis	112	41.6	229	13.1	<0.001
Non-union fracture	86	32.0	325	18.6	<0.001
Compartment syndrome	56	20.8	206	11.8	<0.001
Mechanical complication of orthopedic device	43	16.0	122	7.0	<0.001
Other complication due to device	45	16.7	155	8.9	<0.001
Infection due to internal prosthetic device	71	26.4	172	9.8	<0.001
Cellulitis	97	36.1	291	16.6	<0.001
Post-traumatic wound infection	93	34.6	224	12.8	<0.001
Post-op infection	109	40.5	298	17.0	<0.001
**Pulmonary**					
Pulmonary embolus	27	10.0	48	2.7	<0.001
Pneumonia	27	10.0	84	4.8	<0.001
Pulmonary insufficiency post-trauma	33	12.3	81	4.6	<0.001
Pulmonary collapse	26	9.7	90	5.1	<0.001

Notes: Frequencies represent the number of Service members with at least one of the indicated diagnoses. Abbreviations: limb salvage with secondary amputation (LS-SA), non-threatening limb trauma (NTLT).

## Data Availability

All data supporting the findings of this study are available within the paper.
